# A New Model for Hendra Virus Encephalitis in the Mouse

**DOI:** 10.1371/journal.pone.0040308

**Published:** 2012-07-10

**Authors:** Johanna Dups, Deborah Middleton, Manabu Yamada, Paul Monaghan, Fenella Long, Rachel Robinson, Glenn A. Marsh, Lin-Fa Wang

**Affiliations:** CSIRO Livestock Industries, Australian Animal Health Laboratory, Geelong, Australia; University of Georgia, United States of America

## Abstract

Hendra virus (HeV) infection in humans is characterized by an influenza like illness, which may progress to pneumonia or encephalitis and lead to death. The pathogenesis of HeV infection is poorly understood, and the lack of a mouse model has limited the opportunities for pathogenetic research. In this project we reassessed the role of mice as an animal model for HeV infection and found that mice are susceptible to HeV infection after intranasal exposure, with aged mice reliably developing encephalitic disease. We propose an anterograde route of neuroinvasion to the brain, possibly along olfactory nerves. This is supported by evidence for the development of encephalitis in the absence of viremia and the sequential distribution of viral antigen along pathways of olfaction in the brain of intranasally challenged animals. In our studies mice developed transient lower respiratory tract infection without progressing to viremia and systemic vasculitis that is common to other animal models. These studies report a new animal model of HeV encephalitis that will allow more detailed studies of the neuropathogenesis of HeV infection, particularly the mode of viral spread and possible sequestration within the central nervous system; investigation of mechanisms that moderate the development of viremia and systemic disease; and inform the development of improved treatment options for human patients.

## Introduction

Hendra virus (HeV) causes serious systemic infection with pneumonia and encephalitis in humans, horses and various laboratory animals [1], [2], [3], [4]. It is a single-stranded, negative-sense RNA virus belonging to the family *Paramyxoviridae* and is classified within the genus *Henipavirus* which it shares with one other virus, Nipah virus (NiV). HeV first emerged in the Brisbane suburb of Hendra in 1994, where it caused the deaths of one human and fourteen horses [5]. Since then a further thirty four HeV outbreaks have been identified along the mid to north-eastern coast of Australia with infection of five more humans (of whom three died) and numerous horses [6], [7], [8], [9]. Pteropid bats have been identified as the reservoir host [10], however epidemiological evidence does not support direct bat to human transmission. Horses have been an intermediate host in the transmission of disease to humans in all cases. There are as yet no readily available effective therapies or prophylaxis for HeV infection, either for use in humans or other susceptible animals.

Of necessity, HeV pathogenesis studies and evaluation of vaccine and therapeutic candidates must be carried out in animal infection models under Biosafety Level 4 (BSL4 conditions). Several species have been used for this purpose including: ferrets, hamsters, guinea pigs, pigs, cats, horses, and African green monkeys [3], [11], [12], [13], [14], [15], [16]. With bats, the list comprises species from six orders including; Rodentia, Primates, Chiroptera, Cetartiodactyla, Perrisodactyla and Carnivora. The broad species susceptibility is unusual for a member of the family *Paramyxoviridae* and is attributed largely to the highly conserved nature [17] of the host receptors for the virus, Ephrin B2 and B3 [18], [19]. Despite the possession of relevant receptors [19], the laboratory mouse, a most useful host on account of their small size, ease of handling, and vast library of available reagents, is reported to be resistant to HeV infection and disease [20].

Westbury *et al* in 1995 reported resistance of mice to HeV infection in a study that was designed to identify a suitable laboratory animal model of HeV disease. Juvenile BALB/c mice were inoculated with 5000 median tissue culture infective doses (TCID_50_) of virus by a parenteral route and observed for clinical signs of infection. Mice remained clinically well throughout the 21 day study period and, after euthanasia, there was no evidence of infection by gross or histological examination, virus isolation or serology. Similar results were reported by Wong *et al* in 2003, who investigated the susceptibility of mice to the closely related Nipah virus [21] by inoculating juvenile Swiss brown mice by either parenteral or intranasal routes.

An understanding of the mechanisms of resistance of mice to HeV may provide novel targets for therapeutic and preventative intervention of human infections. Furthermore, circumvention of such mechanisms may induce a useful mouse model of HeV disease. Therefore, in view of the limited previous work, we decided to re-evaluate the apparent resistance of mice to HeV infection by investigating the outcome of HeV exposure by various routes to inbred mice of different ages and strains. Additionally, quantitative real-time polymerase chain reaction (qPCR), a technique not available at the time of the initial studies, would be used for detecting evidence of viral replication.

We found that mice are susceptible to HeV infection when exposed via the intranasal route, but resist infection when challenged by a parenteral route. Infection manifested as acute, transient, and asymptomatic virus replication in the upper and lower respiratory tracts, together with clinically significant encephalitis that has a longer incubation period than is reported for other models of fulminating HeV disease. The pattern of central nervous system involvement (CNS) supports neuroinvasion by the anterograde route (spread from the neuron cell body toward the axon terminus) and, importantly, transneuronal spread within the CNS. Over all, the study demonstrated that mice are susceptible to HeV infection and has provided a new and important model for HeV induced encephalitis.

## Results

### C57BL/6 Mice Develop Clinical Disease After Intranasal but not Parenteral Exposure to HeV

Ten juvenile (8 weeks) and ten aged (12 months) C57BL/6 mice were each divided into two groups and challenged with 50,000 TCID_50_ HeV using either an intranasal or subcutaneous route of exposure and monitored daily for 21 days post infection (DPI).

Weight loss and temperature changes were not observed for any animals beyond expected minor daily fluctuations. Aged and juvenile mice exposed by the subcutaneous route remained clinically well during the period of observation and at euthanasia there was no evidence of HeV infection by histology, immunohistochemistry or qPCR (Table 1). Specific binding antibody to the soluble form of the HeV G glycoprotein (HeV sG) was detected in sera of two juvenile animals only, using a Luminex microsphere assay [22]. Low levels of serum neutralizing antibody was also detected in one of these animals by serum neutralisation test (SNT) (Table 2).

**Table 1:** Clinical outcomes and viral presence in mice challenged with Hendra virus.

Mouse Strain	Age	Mouse Number	ROI	DED	Clinical signs	qtPCR	Les/Ant
C57BL/6	Juvenile	1	IN	21	None	–	−/−
		2	IN	21	None	–	−/−
		3	IN	21	None	–	−/−
		4	IN	21	None	–	−/−
		5	IN	14	Depression,unreactive to stimuli	+	+/+
		6	SC	21	None	–	−/−
		7	SC	21	None	–	−/−
		8	SC	21	None	–	−/−
		9	SC	21	None	–	−/−
		10	SC	21	None	–	−/−
	Aged	11	IN	16	Depression, ataxia, hypersensitivity, tremors	+	+/+
		12	IN	20	Depression, ataxia, hypersensitivity, tremors	+	+/+
		13	IN	21	Depression, ataxia, hypersensitivity, tremors	+	+/+
		14	IN	12	Found dead	ns	+/+
		15	IN	11	Found dead	ns	ns
		16	SC	21	None	–	−/−
		17	SC	21	None	–	−/−
		18	SC	21	None	–	−/−
		19	SC	21	None	–	−/−
		20	SC	21	None	–	−/−
BALB/c	Juvenile	21	IN	21	None	+	+/+
		22	IN	21	None	+	+/−
		23	IN	21	None	–	–
		24	IN	21	None	+	+/+
		25	IN	21	None	+	+/+
	Aged	26	IN	11	Depression, ataxia, hypersensitivity, tremors	+	−/−
		27	IN	17	Prostrate, tremors	+	+/+
		28	IN	21	None	+	+/+
		29	IN	18	Depression, ataxia, hypersensitivity, tremors	+	+/+
		30	IN	21	None	+	+/−

ROI: route of infection; DED: Day of euthanasia or death; IN: intranasal; SC: subcutaneous; qtPCR:real time PCR analysis for viral RNA in brain tissue; Les/Ant: presence of lesions by histology/presence of antigen by immunohistochemistry in brain tissue; ns: no sample; (+) positive; (−) negative.

**Table 2:** Specific (binding) antibody to HeV sG and serum neutralisation titres at euthanasia.

Strain	Age	Mouse #	MFI (BA)	SNT
C57BL/6	Juvenile	1	352	–
		2	***4145***	–
		3	***19321***	–
		4	***23937***	1∶5
		**5**	ns	ns
		6	***444***	–
		7	130	–
		8	***13388***	1∶5
		9	106	–
		10	219	–
	Aged	**11**	***4265***	–
		**12**	***19064***	1∶10
		**13**	***24124***	1∶5
		**14**	ns	ns
		**15**	ns	ns
		16	133	–
		17	120	–
		18	174	–
		19	132	–
		20	108	–
BALB/c	Juvenile	21	***28633***	1∶20
		22	***3440***	1∶5
		23	***19915***	–
		24	***28632***	1∶10
		25	***23262***	1∶5
	Aged	**26**	***1088***	–
		**27**	***24128***	–
		28	***4235***	–
		**29**	***15465***	–
		30	***24685***	1∶10

MFI: median fluorescence intensity; BA: Binding antibody to HeV sG; SNT: serum neutralisation test; Bold: indicates animals that developed clinical disease; Bold italics: indicates a positive antibody response following challenge where a positive result equals an MFI >406.

By contrast, aged animals exposed intranasally were either unexpectedly found dead (mice 14 and 15, 12 and 11 DPI) or developed peracute neurological disease during the study period (mice 11–13) necessitating euthanasia on 16, 20 and 21 DPI. Affected animals showed ataxia, muscle tremors and hypersensitivity. One juvenile mouse (mouse 5) with intranasal exposure also developed a neurological illness requiring euthanasia. Mouse 5 displayed a slower disease onset compared to the aged mice, with a three day waxing and waning depressive illness culminating in severe depression, lack of response to stimuli and hypothermia. In contrast to the systemic vasculitis and multi-organ involvement that are features of HeV infection in other animal species, inflammatory lesions and HeV antigen were only identified in the brain tissue of clinically affected mice and not in heart, lungs, spleen, liver, kidney, ovary, uterus, thymus, pharynx, or mesenteric lymph nodes of any mouse.

**Figure 1:**
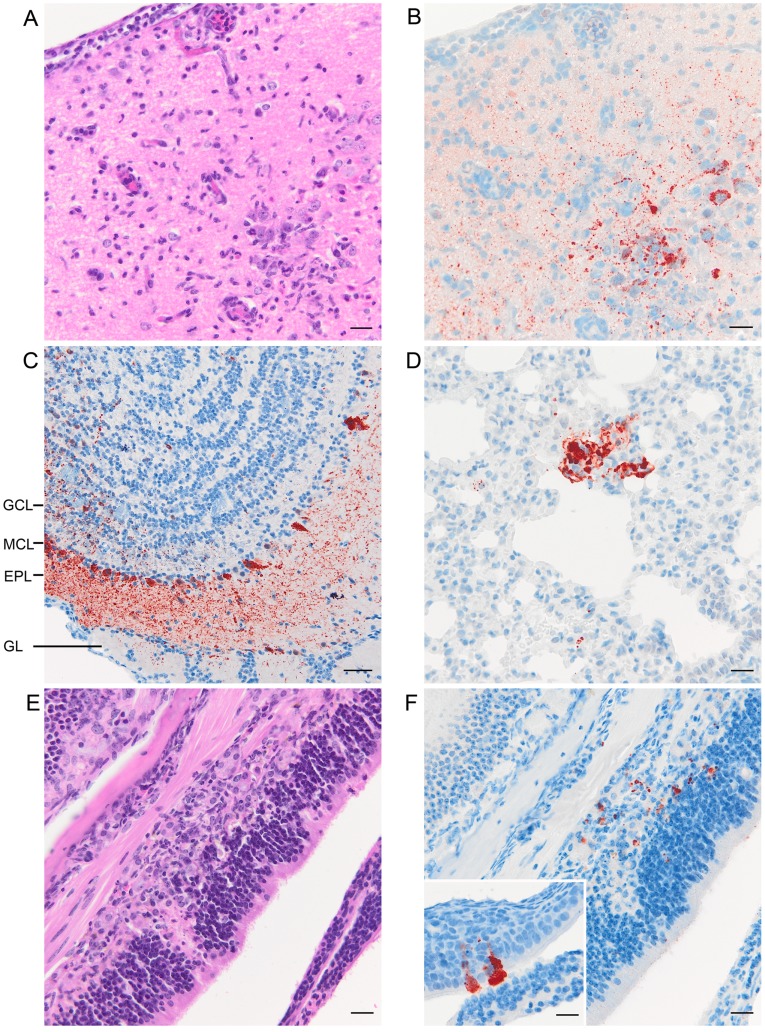
Lesions and antigen staining observed in tissues collected from intranasally challenged aged mice. (A) & (B) Focal encephalitis with microglial activation, glial reaction, perivascular cuffing and a non-suppurative meningitis is seen in the piriform lobe (A)(H&E) and antigen staining (red, IHC) is seen in close association with the lesions (B). (C) Antigen staining (red, IHC) is seen in the glomeruli (GL), external plexiform layer (EPL), mitral cell layer (MCL) and granule cell layer (GCL) of the olfactory bulb. (D) Clusters of antigen positive cells (red, IHC) are seen within bronchoalveolar tissue of the lung. (E) Focal necrotising inflammation is seen in the olfactory mucosa (H&E), and (F) antigen staining (red, IHC) is seen in association with the lesions seen in (E). Inset picture (F) shows antigen staining (red) within cells of the olfactory mucosa (IHC). Scale bars  =  A) 20 µm, B) 20 µm, C) 50 µm, D) 20 µm, E) 20 µm, F) 20 µm and F *inset*) 20 µm.

Encephalitis was confirmed in all five clinically affected mice (Table 1), with meningitis in four of these (mice 5 and 11–13); vasculitis was not identified. Encephalitic lesions were characterised by neuronal degeneration, microglial activation, glial reaction, perivascular cuffing and, where present, non-suppurative meningitis (Figures 1A and 1B). Viral antigen and lesions were detected in the olfactory tract, cortex by the olfactory tract and piriform lobe in each case (Table 3). In mouse 12 lesions were more extensive and included the olfactory bulb, amygdala, thalamus, hippocampus and pons. Except for the pons, all neuroanatomical sites involved are associated with the afferent pathways of olfaction in the brain.

**Table 3:** Distribution of histologic lesions and viral antigen in the brain of intranasally HeV challenged mice.

			Distribution of Lesions^a^									
Age	Strain	Mouse Number	OB	O.Tr	Cortex by O.Tr	PL	Amyg	Hippo	Thalamus	Cortex FL	Pons/Med	Cerebellum
C57BL/6	Juvenile	1										
		2										
		3										
		4										
		5	ns	+/+	+/+	+/+	+/−				ns	ns
	Aged	11	ns	+/+	+/+	+/+	+/−					
		12	+/+	+/+	+/+	+/+	+/+	+/+	+/+	+/+	+/+	
		13	ns	+/+	+/+	+/+	+/+					
		14		+/+	+/+	+/+			−/+		ns	ns
		15^b^										
BALB/c	Juvenile	21	+/+	+/+	+/+							
		22		+/−								
		23										
		24	+/+	+/+	+/+							
		25	+/+							+/+		
	Aged	26										
		27	ns	+/+	+/+	+/+	+/+	+/+	+/+	+/+		
		28	+/+	+/+	+/+	+/+	+/+			+/+		
		29	ns	+/+	+/+	+/+	+/+	+/+	+/+	+/+	+/+	
		30							+/−			

a presence of lesion/presence of antigen

b Samples not available; OB: Olfactory bulb; O.Tr: Olfactory tract; PL: Piriform lobe; Amyg: Amygdala; Hippo: Hippocampus; FL: Frontal Lobe; Med: Medulla; (+) present; (−) not present; empty cells represent −/−; ns: no sample.

**Figure 2:**
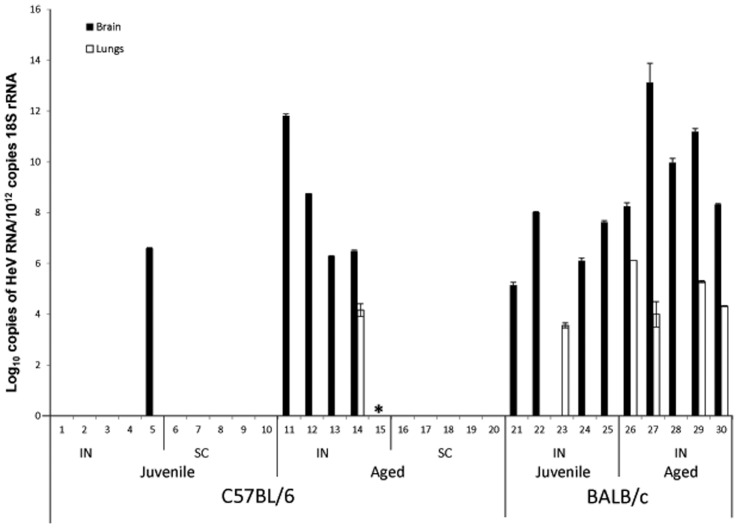
Viral loads in brain and lung tissue of HeV challenged mice. RNA was extracted from tissue samples collected at euthanasia and analysed in duplicate using Taqman PCR assay detecting HeV N RNA and 18S rRNA and expressed as copies HeV(N)/10^12^copies 18S. * samples not available.

In addition, we analysed tissues and whole blood from each animal for the presence of viral genome using qPCR. All mice that developed clinical disease (excluding mouse 15 for which samples were not available) had high levels of viral genome present in brain tissue ranging from 10^6^.^3^ to 10^11^.^8^ HeV copies/10^12^ 18S copies (Figure 2). All other mice were negative for viral genome in brain tissue. All tissues examined by qPCR including; heart, lung, thymus, pharynx, spleen, kidney, ovary, uterus and mesenteric lymph nodes, were negative for viral genome except in two cases; heart tissue of mouse 5 and lung tissue of mouse 14. In both cases C_T_ values were high at 38.8 and 38.3 respectively, indicating low levels of genome and nearing the cut off for a positive sample set at a C_T_ of 39.6. It is of interest to note that mouse 14 died comparatively early in the course of the study (day 12) and the viral RNA detected in the lungs was consistent with the results of mice that were euthanased in the earlier phases of the subsequent time course trial (described below). Low levels of viral genome (C_T_ 39.2) were also detected in blood samples from one juvenile intranasally exposed mouse, mouse 3. Whether viral genome detected in the above three cases reflected replication at the time of euthanasia or residual genome from earlier transient infection could not be determined.

Virus reisolation was attempted on all tissues positive for viral RNA by qPCR with a C_T_ value less than 39.6. Virus isolation was negative for all tissues assayed including brain tissue where high levels of viral genome, antigen, and lesions were detected with and without neutralizing antibody.

All animals that developed clinical disease were positive for a binding antibody response to HeV sG by Luminex assay. Neutralising antibody was also detected in two of these animals by SNT, however titres were evidently insufficient to control intracranial infection. In general, neutralising antibody titers were considerably lower than those reported in convalescent horses following field HeV infection [23].

### HeV Infection of Mice is not Mouse Strain Dependent

We wished to ascertain whether the aforementioned outcomes of exposure were restricted to the C57BL/6 mouse strain. Accordingly, the intranasal component of the above study was repeated in the widely used BALB/c strain. Five juvenile and five adult BALB/c mice were challenged with 50,000 TCID_50_ HeV via the intranasal route and monitored daily for 21 days.

Clinical outcomes observed for BALB/c strain mice exposed to Hendra were generally similar to those observed for C57BL/6 mice. As with C57BL/6 mice, weight loss and temperature changes were not observed. All five juvenile mice remained healthy throughout the trial period, whereas three of the five aged mice (mouse 26, 27 and 29) developed clinical neurological disease necessitating euthanasia on 11, 17 and 18 DPI (Table 1). Examination of tissues by histology and immuno-histochemistry revealed similarities and also differences to findings for C57BL/6 mice. In contrast to C57BL/6 mice, lesions and viral antigen were detected in nervous tissues of both symptomatic and asymptomatic animals (Table 1). Two of the three animals that developed clinical disease had encephalitis and viral antigen in the brain: in the third animal (mouse 26) the olfactory bulb was not sampled, and only half the brain was available for histological assessment (the rest was used for qPCR and virus isolation). Four asymptomatic mice (one aged and three juvenile) had encephalitis and viral antigen in brain, with a further two asymptomatic, mice having encephalitis only. Encephalitic lesions and viral antigen deposition in BALB/c mice were also largely confined to neuro-anatomical sites associated with afferent pathways of olfaction (Table 3). The lesions were generally more extensive in aged animals and, while this likely accounts for the differences in clinical signs observed here between old and young mice, it raises the possibility that following longer periods of observation illness may develop in young mice.

Viral genome was detected in brain tissue of all young and aged BALB/c mice, apart from one juvenile mouse in which neither antigen nor lesions were identified (Figure 2). Viral genome loads in brain ranged from 10^5.1^–10^13.1^ copies HeV/10^12^ 18S; viral genome was also present in lung tissue of four of the five aged animals and one of the five juvenile animals (Mouse 23, Figure 2), albeit at lower levels than seen in brain tissue.

Genome was also detected in pharyngeal tissue of one aged mouse (#29) at very low levels (C_T_ 38.9), without lesions or HeV antigen by histology and immunohistochemistry. As with the C57BL/6 mice, virus could not be isolated from any samples including brain where high levels of viral genome, antigen, and lesions were detected and often in the absence of neutralizing antibody.

Specific antibodies to HeV sG were detected in all mice by Luminex assay and neutralising antibody was detected in five of the ten animals (see Table 2). Again, SNT titres were low l (1∶5 to 1∶20) and did not correlate with clinical outcome.

### Aged Mice are More Susceptible to Clinical Disease than Juvenile

The above results suggested that aged mice were more susceptible to clinical disease following intranasal HeV exposure than juvenile animals. Using logistic regression analysis to determine if this trend was significant we found that 80% of aged animals developed clinical disease (s.e.  = 12.7%) and only 10% of juvenile animals (s.e.  =  9.5%) suggesting a real difference (70%, s.e.  =  15.8%) in susceptibility to the development of disease between the aged and juvenile animals (p<0.01).

### HeV Causes a Transient Lung Infection

The study investigating the susceptibility of BALB/c strain mice to HeV infection described above revealed that four out of five aged mice had viral RNA in lung tissue as late as 21 DPI in the absence of clinically apparent respiratory disease or detectable pulmonary pathology at euthanasia. In light of these findings, a time-course study was performed to evaluate whether infection of the upper and lower respiratory tracts developed in aged mice following HeV exposure. Aged BALB/c mice exposed to HeV by the intranasal route were euthanased at each time point of the following schedule; two mice every 48 hours from 1-14 DPI, two mice every 72 hours from 15 to 23 DPI, and three mice at 28 days PI, or on development of clinical disease according to pre-determined humane endpoints defined from the previous study. Whole brain, lung, nasal turbinates and other tissue samples were examined by histology and immunohistochemistry. Lung tissue was further analysed for presence of viral genome and live infectious virus by qPCR and virus isolation, respectively (Table 4).

**Table 4:** Clinical outcomes and viral presence in selected organs of aged BALB/c strain mice, challenged with Hendra virus via the intranasal route.

Mouse #	DPI	CSx	Lungs			Nasal Mucosa	Brain
			Hist/IHC	vRNA	VI	Hist/IHC	Hist/IHC
101	2	–	−/−	+	–	–	−/−
102	2	–	−/−	+	–	–	−/−
103	4	–	−/−	+	+	–	−/−
104	4	–	−/−	+	+	–	−/−
105	6	–	−/+	+	+	−/+	−/+
106	6	–	−/+	+	+	−/+	−/+
107	8	–	−/+	+	+	–	+/+
108	8	–	−/+	+	+	+/+	−/+
109	9	+	−/+	+	+	+/+	−/+
110	10	+	ns	+	–	+/+	+/+
111	10	+	−/+	+	+	–	+/+
112	12	–	−/−	+	–	–	+/+
113	12	–	−/−	+	–	–	+/+
114	14	–	−/+	+	–	–	+/+
115	14	–	−/−	+	–	+/+	+/+
116	16	+	−/−	–	–	–	+/+
117	17	–	−/−	+	–	−/+	+/+
118	17	–	−/−	–	–	–	+/+
119	17	+	−/−	–	–	–	+/+
120	18	+	−/−	–	–	ns	+/+
121	20	–	−/−	–	–	–	+/+
122	23	–	−/−	–	–	–	+/+
123	28	–	−/−	–	–	ns	+/+

DPI: days post infection at euthanasia; CSx; Clinical signs; Hist/IHC: Histologic lesions/Antigen presence by immunohistochemistry; vRNA: viral RNA detected by real time PCR analysis; VI: Virus Isolation;(−) negative; (+) positive; ns:no sample.

Mice sampled between days 6 and 14 pi were positive for HeV viral antigen in bronchoalveolar tissue, but antigen deposits were both dense and focal and it was not possible to distinguish on morphologic grounds whether alveolar lining cells, alveolar interstitium, or endothelial cells were involved. Live virus was reisolated from the lungs of mice sampled between 4 and 10 DPI (Table 4) consistent with transient infection of the lower respiratory tract after intranasal exposure to HeV. The viral titres detected were comparatively low with a maximum titre (10^2.3^ TCID_50)_ recovered from a mouse on day 6. Interestingly, HeV antigen deposition in lung (Figure 1D) was not associated with detectable bronchoalveolitis and pulmonary vasculitis was not identified. In the upper respiratory tract, immunopositive cells - sometimes accompanied by small focal necrotising inflammatory lesions (Figure1E and 1F), were detected in olfactory mucosa of mice between 6 to 17 DPI. It was not determined whether cells involved were supporting cells, basal cells or olfactory sensory neurons.

### Mice Develop Encephalitis Following HeV Exposure without Detectable Viremia or Significant Systemic Involvement

Vasculitis and subsequent multi-organ infection is a feature of all reported animal models of HeV. In the initial observational study above there was little evidence of HeV virus spread to tissues other than brain apart from low levels of genome recovered from lung in some animals. To assess the extent of systemic involvement outside the respiratory tract, all other tissues for each animal collected in the time-course study were analysed for lesions and viral antigen/genome.

All tissues examined apart from the aforementioned nasal mucosa, lung, and brain were negative for both lesions and antigen. In brain, immunopositive cells were first detected 6 DPI (Table 4), and from 8 DPI lesions characterised by neuronal degeneration, microglial activation, glial reaction, perivascular cuffing and non-suppurative meningitis were also identified. Vasculitis was not detected in any animal.

All other tissues excluding brain, which was fixed for histology and immuno-histochemistry in its entirety, were assessed for viral genome by qPCR and virus isolation was attempted from any positive samples. Some tissues, notably thymus that likely incorporated cranial mediastinal lymph node, were positive for viral genome (Table 5), although negative for viral antigen and lesions. On occasion the relative genetic load was comparable to that seen in lung tissues although virus was not reisolated from any PCR positive tissues other than lung.

**Table 5:** Viral RNA loads in tissues at euthanasia.

DPI	Mouse #	Log_10_ HeV RNA copies/10^12^ 18S rRNA										
		Lung	Heart	Kidney	Liver	Mes LN	Spleen	Pharynx	Thymus	Uterus	Ovaries	Cerv LN
2	101	3.80										
	102	4.15			1.68			3.70				
4	103	6.85*										
	104	9.05*										
6	105	6.21*	4.42	2.93	3.64							
	106	7.82*					3.53					
8	107	6.75*							2.83			2.67
	108	6.91*										2.49
9	109	8.11*							5.69			
10	110	6.94	1.57			2.96			2.46	1.19	1.17	
	111	4.75*							6.07			
12	112	6.63							4.95			
	113	6.19										
14	114	4.33	2.55			3.15						2.08
	115	3.52										
16	116											5.63
17	117	5.37							4.65			
	118											
	119					5.67			5.76			
18	120									4.40		
20	121	6.62										
23	122											4.97
28	123									6.30		

DPI: day of euthanasia post infection; Mes LN: mesenteric lymph nodes; Cerv LN: cervical lymph nodes;

(*) virus isolation positive; empty cells indicate a negative result.

For assessment of transient viremia, blood samples were also collected at euthanasia from each animal in the time-course study and RNA extracted using a Ribopure blood extraction kit. Previous work showed this extraction method to be the most sensitive for qPCR detection of HeV RNA in experimentally infected horses (A. Foord, personal communication). In mice, a low level (C_T_ of 39.2) of viral RNA was detected in blood from only one animal (#111); this level of genome was very close to our cut off of C_T_ of 39.6 (equivalent to 1 copy RNA from the standard curve).

### HeV Viral Antigen and Lesions in the Brain were Largely Confined to Neuroanatomical Sites Associated with the Afferent Olfactory Pathway

As viremia and systemic spread was not an important feature of HeV in mice, neuroinvasion was unlikely to have been mediated via the haematogenous route. To better characterise the route of neuroinvasion, the distribution of viral antigen and lesions within the brain were examined in more detail. Brains collected at each point of the time-course study were transversely sectioned at 2 mm intervals; paraffin embedded sections were stained with haematoxylin and eosin for histology and with polyclonal anti-HeV N antibody for detection of HeV antigen (Table 6 *)*.

**Table 6:** Distribution of histologic lesions and viral antigen in the brain of intranasally HeV challenged mice.

Mouse #	DPI	CSx	Distribution of Lesions^a^								
			OB	OT	PL	Amyg	Hippo	Thal	Hypo	Med/Pons	VN
101	2	–									
102	2	–									
103	4	–									
104	4	–									
105	6	–	−/+								
106	6	–	−/+								
107	8	–	+/+		−/+						
108	8	–	−/+								
109	9	+	−/+		−/+						
110	10	+	+/+								
111	10	+	+/+	+/+		+/+					
112	12	–	+/+		+/+	+/+					
113	12	–	+/+		+/+	+/+					
114	14	–	+/+		+/+						
115	14	–	+/+		+/+						
116	16	+	+/+		+/+	+/+		+/+		+/+	+/+
117	17	–	+/+		+/+						
118	17	–	+/+		+/+		+/+				
119	17	+	+/+	+/+	+/+	+/+	+/+	+/+			
120	18	+	+/+	+/+	+/+	+/+		+/+	+/+	+/+	
121	20	–	+/+		+/+		+/+				
122	23	–	+/+								
123	28	–	+/+								

a presence of lesion/presence of antigen; DPI; Days post infection; CSx: Clinical signs; OB: Olfactory bulb; OT: Olfactory tubercle; PL: Piriform lobe; Amyg: Amygdala; Hippo: Hippocampus; Thal: Thalamus; Hypo: Hypothalamus; Med/Pons: Medulla/Pons; VN: Vestbular nuclei; (+) present; (−) not present; empty cells indicate neither lesions nor antigen detected.

HeV antigen was first detected in clinically healthy mice euthanased on day 6 and was located in the olfactory bulb involving periglomerular cells, mitral cells, granule cells and associated cell processes (Figure 1C). By day 8 there was histological evidence of an inflammatory response closely associated with immunopositive cells. On 9 and 10 DPI three mice showing clinical signs of disease were electively euthanased. In addition to antigen in the olfactory bulb of the brain, two of these mice had antigen detectable in the piriform lobe, olfactory tubercle and amygdala which are all components of the primary olfactory cortex and are connected with each other via synapses. Clinically healthy mice euthanased between days 12 and 20 post infection were uniformly positive for antigen and also inflammatory lesions in the olfactory bulb and piriform lobes. Occasionally, lesions and antigen were seen in the amygdala and more caudally in the hippocampus. The latter feature was especially seen in those mice euthanased in the latter part of the time-course study (day 17 onwards); both of these anatomic structures are associated with the afferent pathways of olfaction in the brain.

Three mice developed clinical disease between days 12 and 20 pi, one each on days 16, 17 and 18. As seen in Table 6, lesions and antigen detected in the brains of these mice were more extensive than for the animals that did not develop clinical disease. In diseased mice, lesions and antigen were not only detected in the common structures described above, but also in higher processing structures of the cortex such as the thalamus and hypothalamus. Interestingly, two of these mice, mouse 116 and 120, were positive for antigen and lesions in the medulla and pons, and in mouse 116 the lesions and antigen were found in the vestibular nuclei proximal to the vestibulocochlear nerve.

Mice 122 and 123, euthanased 23 DPI and 28 DPI respectively, were clinically healthy. In these mice, lesions and antigen were confined to the olfactory bulb.

### Viral Antigen is Largely Restricted to Neuronal Cells within the Brain

**Figure 3:**
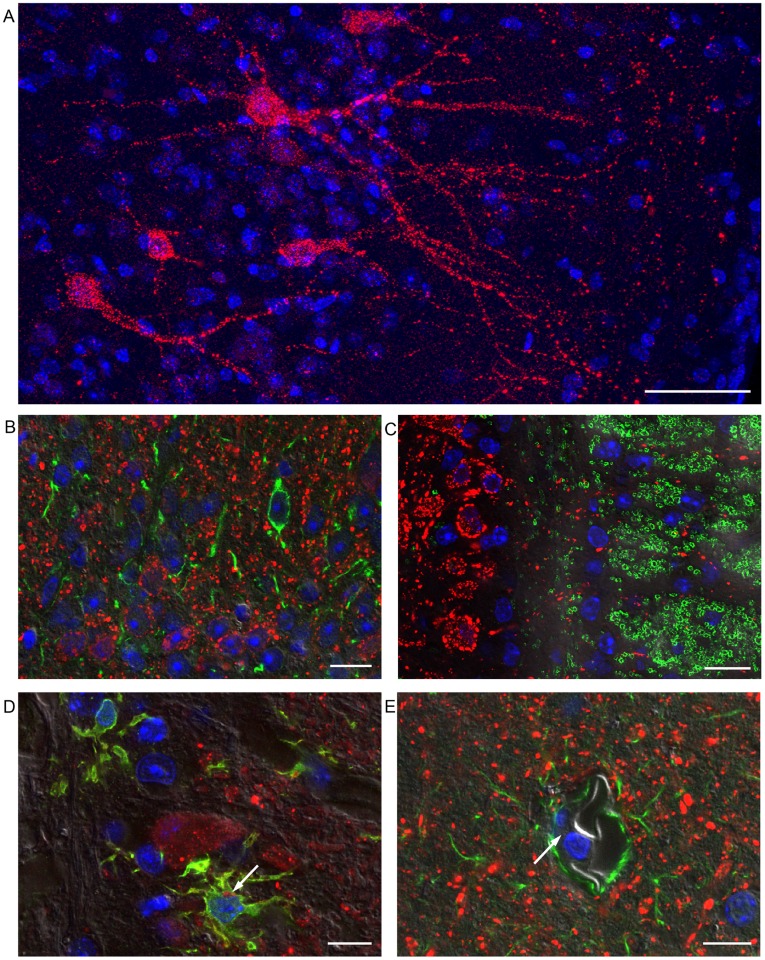
Representative immunoflourescent confocal images of vibrating microtome sections of brain tissue from HeV infected mice. (A) HeV antigen (red) was detected in cells of distinctly neuronal morphology at day 11 post infection (PI) in the piriform lobe. (B) Viral antigen (red) was not seen in cells identified as astrocytes through positive staining for GFAP (green) in olfactory bulb at day 9 PI. (C) HeV antigen (red) was not seen in cells identified as oligodendrocytes through positive staining for MBP (green) in olfactory bulb at day 11 PI. (D) HeV antigen was only occasionally seen in microglia, identified with labelling for Iba1 (green) in olfactory bulb at day 9 PI. HeV antigen labeling (arrow) was discrete and circumscribed, consistent with its presence within a cellular compartment such as a lysosome. (E) Section of olfactory bulb showing a capillary amongst HeV infected cells at day 10 PI. HeV antigen (red) and GFAP (green) are not colocalised. The endothelial cell cytoplasm (arrow) is negative for HeV antigen. Scale bars  =  A) 50 µm, B) 10 µm, C) 15 µm, D) 10 µm and E) 10 µm.

As encephalitis was a major feature of Hendra infection in mice, co-localisation studies were performed using confocal imaging to identify affected cell types within the brain. Perfused brains were collected from three aged BALB/c mice, on DPI 9, 10, and 11 and stained for HeV antigen. Antigen was seen in brain sections from all three mice and with a similar distribution to that observed by immunohistochemistry. Antigen appeared to be present in cells with neuronal morphology only (Figure 3A). In order to confirm this observation, sections were labeled with antibodies recognizing neurons (NeuN and NFP1), astrocytes (GFAP) microglia (IBA1) and oligodendrocytes (MBP). Dual labeling of sections of infected mice with these markers and with HeV antibodies indicated that there was no co-localisation of Hendra antigen with GFAP or MBP (Figure 3B and 3C). On rare occasions there was evidence of low intensity staining for Hendra viral antigen and microglial markers in single cells (Figure 3D). The Hendra protein labelling appeared to be discrete and circumscribed which would be consistent with its presence within a cellular compartment such as lysosomes. Compared with uninfected control tissue, there was reduced labeling of neurons with neuronal markers (NeuN and NFP1) in infected tissue. Therefore, we could not use co-localisation studies with these antibodies to confirm HeV antigen in neurons. In order to explore whether viral proteins could be identified within endothelial cells of capillaries or larger vessels, differential interference contrast (DIC) images were taken of areas of Hendra replication. The capillary lumen and endothelial cell cytoplasm were clearly identified and in no case was Hendra antigen detected in the cytoplasm of endothelial cells (Figure 3E).

Taken together, this data suggest that HeV replication is restricted to neurons during infection of the brain.

## Discussion

This paper reports the successful infection of mice following intranasal exposure to HeV, with reliable induction of viral encephalitis in aged mice of two strains. A previous investigation by Westbury *et al* [20] reported that juvenile immunocompetent BALB/c mice exposed to HeV by a parenteral route did not develop clinical or pathological signs of infection although intracranial inoculation of suckling mice had been uniformly lethal (G. Crameri, personal communication). Infection studies were not pursued further and up to now a mouse model of HeV infection has not been available.

We have now confirmed progressive involvement of the mouse CNS by HeV which is most likely established via a non-haematogenous route of neuroinvasion, as has been established or proposed for other paramyxoviruses including Sendai virus in mice [24], Canine distemper virus in ferrets [25], and the closely related Nipah virus in pigs [26].

Our data are consistent with anterograde HeV entry into the brain, possibly via olfactory sensory neurons (OSN). Viral antigen was detected in both the olfactory mucosa and the olfactory bulbs of intranasally challenged mice as early as day 6 post infection and these two structures are connected by olfactory sensory neurons (OSN). OSNs are located within the olfactory mucosa of the nasal cavity and project cilia out into the lumen of the nasal cavity to detect odorants [27]. Their axons project to the olfactory bulb of the brain [28] and thereby provide pathogens with a potential route of direct transmission from the nasal cavity to the brain. Within the olfactory bulbs OSNs synapse with mitral, tufted (projection neurons) and peri/juxtaglomerular cells (interneurons) in the glomeruli [29], and secondary dendrites of the mitral and tufted cells synapse with dendrites of granule cells, which reside largely in the granule cell layer [30]. The trigeminal nerve may provide another possible route of neuroinvasion from the nasal cavity to the glomeruli of the olfactory bulbs. Peripheral peptidergic fibres of the trigeminal nerve innervate the olfactory epithelium and at least some of these fibres will send collaterals to the olfactory bulb (fibres terminate in the glomeruli) [31] en route to the trigeminal ganglion and the contribution of these fibres to viral invasion of the olfactory bulbs requires closer investigation. Viral antigen and lesions were also identified in the pons and medulla in four mice. We suggest that in these cases brain infection occurred via another peripheral neural pathway that enters the CNS at the brainstem such as the trigeminal [27] or the vestibulocochlear nerve [32] and it is of interest that in one animal antigen was localised to the vestibular nuclei of the medulla, which would be consistent with neuroinvasion of the vestibulocochlear nerve [32] after infection arising in the inner ear.

The confinement of HeV antigen to neurons of mice, and the pattern and time course of viral antigen and lesions detected in their olfactory glomeruli, mitral and granule cell layers of the olfactory bulbs, as well as deeper structures in the olfactory pathway (such as elements of the olfactory cortex including the olfactory tubercles, piriform lobe and amygdala [33], and then onto the hippocampus, thalamus and hypothalamus [27]) suggests that HeV uses a transneuronal mode of spread within the murine brain that is mediated via synaptic connections. Transneuronal spread via synapses has been described for both H5 influenza virus [34] and Herpes simplex virus type 1 [35].

Transneuronal spread via synapses using cell contact dependent processes without budding of infectious virions may explain how HeV could not be reisolated from infected mouse brains, despite the presence of encephalitis, viral antigen and recovery of substantial viral genome. Transneuronal virus movement within the brain using cell contact dependent processes at the synapse without budding of infectious virions or syncytial cell formation has been proposed for another member of the family Paramyxoviridae, measles virus [36].

In humans, encephalitis represents a significant complication of HeV infection. Of the seven persons known to have contracted Hendra infection three have died from encephalitis and a fourth developed encephalitis and recovered [1], [4], [37]. Of the three patients that succumbed to encephalitis one case was a recrudescent infection, occurring 13 months after the acute disease [38]. Meningoencephalitis is also an important feature of the natural infection in horses, where it may persist weeks into convalescence, and is also a feature of other experimental infection models [2], [3], [39]. The pathogenesis of HeV infection of the central nervous system (CNS), including the means of spread throughout the brain and the mechanism of recrudescence, is poorly understood.

The mouse model of HeV encephalitis lends itself well to pathogenesis studies of this type as the animals do not succumb to fulminating systemic infection prior to the establishment of significant neuropathology, the CNS lesions develop over a sufficiently long period of time that their progression can be monitored in considerable detail, and high levels of genome persist in brain in spite of virus neutralizing antibody, albeit that it occurs at low level. A particular advantage is that mice are small and comparatively easy to handle at BSL4 and there is an established library of available reagents for neuropathogenetic studies. HeV infection has also been established in two strains of mice, BALB/c and C57BL/6, providing investigators with the opportunity to employ transgenic mouse systems on two genetic backgrounds to examine disease processes, particularly with regard to the immune response in CNS infection.

Although at present there is insufficient data available from human HeV cases to conclude whether neuroinvasion by the olfactory sensory neuron pathway is of primary pathogenetic importance in this species, we do note that the most likely route of human infection is contact droplet infection of the nasopharynx, and so exposure by this route is plausible. More importantly, the pattern of spread of HeV through the brain of mice is best explained by an ability of the virus to employ direct trans-neuronal transmission without concomitant generation of an infectious virion: these observations are of key pathogenetic significance to the human disease. Not only do they provide a possible explanation for failure to reisolate HeV from both sub-acute (I. Smith, unpublished data) and recrudescent [38] human cases of HeV using conventional techniques and in whom there was ample evidence of its ongoing replication, they raise important challenges to optimizing therapeutic interventions.

A significant difference in response to infection is seen between aged and juvenile mice where aged mice appear to have a greater propensity to develop clinical disease compared with juvenile animals. A similar trend has been noted for other viral infections of mice, most notably SARS infection. In this case aged mice were susceptible to clinical disease development after challenge with SARS while their juvenile counterparts were not [40]. This observed difference in response to HeV infection between juvenile and aged mice can be used to model and investigate the mechanisms whereby age may affect clinical outcome.

This model may be of particular value in studying aspects of recrudescence. In most other animal models of HeV infection, exposure rapidly leads to acute and fatal disease. In juvenile mice, we have observed that clinical disease may not develop (up to at least day 21 post exposure) despite strong evidence of viral replication in brain that is also continuing in the face of circulating antibody to G protein. Furthermore evidence of encephalitis in these animals was observed in the absence of vasculitis. Some of these characteristics are a feature of both recrudescent human HeV [1] and also NiV [41], [42] infection; the mouse HeV model could be employed to explore the mechanisms by which the potential for virus replication may be sustained in convalescent individuals.

The HeV mouse infection model also provides an opportunity to study mechanisms of viral suppression. Our studies show that although intranasally exposed mice develop transient respiratory infection, the low viral titres detected suggest that infection is controlled in its early phase and cleared. Furthermore, this respiratory infection does not progress to a significant systemic disease as occurs in other animal species [2], [3], [13], [15]. Suppression of infection in the absence of evidence for a robust neutralising adaptive immune response suggests an important role for the mouse innate immune system in this process. HeV has been shown to employ various strategies to evade clearance and control by the innate immune system [43] and we suggest that in the mouse such strategies are rendered ineffective. It will be important in future research to elucidate the mechanism by which mice eliminate respiratory infection and resist systemic disease in order to guide the development of therapeutics that might mimic the process.

In summary, this paper reports three novel observations. Firstly, mice are susceptible to HeV infection after intranasal exposure with aged animals reliably developing encephalitic disease. Their small size and ease of handling, as well as the vast range of biological reagents available for use in the species, render them highly valuable infection models for HeV encephalitis. Secondly, our data strongly support a role for not only anterograde neuroinvasion along sensory neurones but also transneuronal viral spread of HeV within the brain. This finding is of particular importance as infection may be established within the nervous system before current systemic treatments are able to be delivered across the blood-brain barrier. Lastly, mice are resistant to systemic vasculitis and fulminating HeV disease, the mechanisms of which can be explored with therapeutic potential. These findings provide a significant contribution to the body of knowledge in the field and have opened up new areas of investigation, from which significant understanding and further research can arise of benefit to humans as well as animals.

## Methods

For all studies reported in this paper, the animal husbandry and experimental design were approved by the CSIRO Australian Animal Health Laboratory’s Animal Ethics Committee. All animal experimentation was conducted following the Australian National Health and Medical Research Council's Australian Code of Practice for the Care and Use of Animals for Scientific Purposes.

### Animal Handling and Husbandry

Ten aged (12-13 months) and ten juvenile (7-8 weeks) C57BL/6 mice and thirty-one aged (10-13 months) and five juvenile (7-8 weeks) BALB/c mice were used for challenge experiments. Mice were housed in groups of four to five according to age, in cages in a room at BSL4. Animals were fed once daily with complete mouse chow and provided with water *ad libitum*. Animals were allowed 7 days to acclimatise before challenge and were implanted with a subcutaneous temperature chip 5 days prior to challenge. For this procedure and all others requiring restraint for manipulations, an intraperitoneal injection of ketamine (75mg/kg; Ketamil; Ilium, Smithfield, Australia) and medetomidine (10mg/kg; Domitor; Novartis, Pendle Hill, Australia) was used to induce anaesthesia. Anaesthesia was reversed by intraperitoneal administration of atipemazole (1mg/kg; Antisedan; Novartis). Animals were monitored daily at which time temperature, weight and clinical data were collected and recorded. Staff wore fully encapsulating suits with an external air supply and all work with live virus was carried out at BSL4.

### Susceptibility Studies in C57BL/6 Mice

Mice were anaesthetised and exposed to a low passage clinical isolate of HeV (Hendra virus/Australia/Horse/2008/Redlands) [44] by either the intranasal or subcutaneous route. For the intranasal route, five aged (12 months) and five juvenile (8 weeks) mice were anaesthetised, placed in dorsal recumbency and exposed to 50,000 TCID_50_ in 50 µl saline by slow drop application to the nares. For the subcutaneous route, five aged and five juvenile mice were anaesthetised and exposed to 50,000 TCID_50_ virus in 200 µl saline by subcutaneous injection. Animals were monitored daily thereafter and euthanased when reaching a previously determined end-point or at 21 days post infection (pi). The humane end-point was defined as a constant weight loss recorded over 3 days or reaching a 20% loss of pre-infection weight, and/or clinical signs consistent with neurological involvement including ataxia, tremors, depression and behavioural changes. As clinical signs of Hendra infection have not been observed in mice previously, the pre-determined endpoint was modelled on infection outcomes recorded for guinea pigs.

### Susceptibility Studies in BALB/c Mice

The intranasal component of the above study was repeated in five aged (12 months) and five juvenile (8 weeks) BALB/c strain mice.

### Time Course Study

Aged (10–13 months) BALB/c strain mice were challenged with 50,000 TCID_50_ of HeV via the intranasal route. Mice were euthanased and samples collected every second day from days 2–14 pi, every 3 days from days 17–23 pi and, lastly, on day 28 pi; at least one replicate was used for each time point in case mice were lost from the study for reasons not associated with HeV, with three mice being sampled on day 28. If mice reached a pre-determined humane endpoint prior to the scheduled sampling day they were euthanased and samples collected and processed as for animals euthanased according to the schedule.

### Neuroinvasion Study (Confocal Microscopy)

Three aged (12–13 months) BALB/c strain mice were exposed to 50,000 TCID_50_ of HeV using the intranasal route described above. Deeply anesthetised mice were perfused with paraformaldehyde on 9, 10, and 11 DPI for confocal analysis of the brain.

### Sample Collection and Processing

At the end of the study, animals were anaesthetised and blood was collected via cardiac puncture, placed into EDTA and serum separator tubes. Following terminal exsanguination brain, heart, lungs, liver, kidney, pharynx, mesenteric lymph nodes, ovaries, uterus, spleen and thymus tissues were collected. For virus isolation and molecular studies samples were placed into 750 µl viral transport media [PBS with 1% bovine serum albumin and 1x antibiotics (Anti-anti, Invitrogen)] with 250 µl aluminium silicate beads (Biospec Products Inc., Bartlesville, OK, USA). The remaining tissues were fixed in 10% neutral buffered formalin for 48 hours prior to routine processing for histology and immunohistochemistry, the latter using a rabbit polyclonal antibody raised against the NiV N protein [45]. Nasal turbinate and oral swab samples were also collected at euthanasia, swabs were placed into 750 µl viral transport media. Tissue samples were homogenised and aliquots removed for RNA extraction and the remainder was stored at −80°C for subsequent processing.

For the time course study, the brains of all animals were fixed in their entirety and not sampled for virus isolation and molecular studies. Blood was collected via cardiac puncture and 500 µl whole blood placed into 1.3 ml RNAlater for Ribopure RNA extraction. Otherwise sampling was as described above.

For HeV antigen localisation in brain cell studies, animals were deeply anesthetised and euthanased by perfusion with paraformaldehyde to achieve optimal fixation of tissues for confocal microscopic studies. The thoracic cavity was opened and the right auricle of the heart removed. Three ml saline was injected into the left ventricle of the heart to achieve exsanguination. Following saline injection, 11 ml of 4% paraformaldehyde (volume/volume) was slowly injected into the left ventricle of the heart, perfusing the entire animal. Following perfusion, mice were placed under 4% paraformaldehyde soaked tissues and allowed to fix for 1 hour. After initial fixation, the brain was removed and immersion fixed in 4% paraformaldehyde (volume/volume) for a further 24 hours after which it was placed into PBSA for processing.

### RNA Extraction and Quantitation PCR (qPCR) Assay

For RNA extraction, homogenised tissue samples or swab samples from studies 1-3 were centrifuged at 16 000 g for 2 minutes to pellet debris and 100 µl supernatant was mixed with 265 µl MagMAX Lysis/Binding solution (Ambion, Victoria, Australia) and removed from BSL4. RNA was extracted using the MagMax-96 viral RNA isolation kit (Ambion). For the susceptibility studies, 100 µl of EDTA blood was mixed with 265 µl MagMAX Lysis/Binding solution (Ambion, Victoria, Australia) and RNA extracted as described above. For the time course study, RNA was extracted from whole blood mixed in RNAlater using the Mouse RiboPure-Blood RNA Isolation Kit (Ambion). TaqMan qPCR was performed using the AgPath-ID one-step reverse transcription-PCR kit (Applied Biosystems, Victoria, Australia), targeting the N gene of HeV as previously described [46]. Positive results were defined by a cycle threshold (C_T_) value of <39.6 based on a standard curve where this C_T_ represented one copy of target RNA. All samples were normalised against the housekeeping gene 18S and expressed as copy numbers HeV/10^12^ copy numbers of 18S.

### Virus Isolation

Virus isolation was performed on all HeV RNA positive samples detected in the HeV Taqman assay using the standard Vero cell line maintained in our institute and originally obtained from the American Tissue Culture Collection (ATCC) [10], [39]. Supernatants from the homogenised samples were inoculated onto Vero cell monolayers and scored positive if syncytia were present after 4 and 5 days.

### Measurment of Antibody to Soluble HeV G Glycoprotein (HeV sG)

Sera collected both prior to challenge and at euthanasia were analysed for binding to HeV sG protein using a Luminex microsphere assay. Assays were performed on a Bio-Plex Protein Array System (Bio-RadLaboratories, Inc., CA, USA) as previously described [22]. Bio-Plex Manager Software (v 4.1) (Bio-RadLaboratories, Inc., CA, USA) was used for data acquisition and analysis. All samples were assayed simultaneously for each mouse strain. A strong positive control was assayed alongside all samples as was a negative control. A positive result was defined as samples with a median fluorescence intensity (MFI) greater than the mean of all pre-challenge samples plus 3 x the standard deviation, MFI >406.

### Serum Neutralisation Test

Sera collected at euthanasia were gamma-irradiated to inactivate virus. Sera were serially-doubly diluted in a 96 well plate (final volume 50ul/well) to which 200 TCID_50_ HeV was added and incubated for 1 hr at 37°C. Following incubation, 2x10^4^ Vero cells/well were added and the assay read after 3 days incubation at 37°C with 5% CO_2_.

### Confocal Imaging

Whole brains, collected and fixed in 4% paraformaldehyde were immunolabelled as previously described [47]. In brief, they were sectioned at 50 µm thickness with a Leica Vibrating Microtome and stored in PBS at 4°C. Sections were treated with 0.1% Triton X-100 (Sigma-Aldrich) in PBS for 1 hour and blocked with 0.5% Bovine serum albumin (Sigma-Aldrich) in PBS (PBS/BSA) overnight at 4°C. Primary antibodies were diluted in 0.5% PBS/BSA and incubated on sections for 2 hours at 37°C. Antibodies and dilutions were: rabbit anti-IBA1- microglia marker (WAKO) 1∶50, Chicken anti-GFAP-Astrocyte Marker (Abcam [Sapphire Biosciences]) 1∶50, Rat anti-Myelin Basic Protein-Oligodendrocyte Marker (Abcam [Sapphire Biosciences]) 1∶50 and Rabbit anti-HeV N (Australian Animal Health Laboratories [AAHL]) 1∶600. Sections were washed 3 times for 5 minutes with PBS. Mouse Ig Blocking Reagent (Vector Laboratories) was used with mouse primary antibodies: anti-Human Neurofilament protein (DakoCytomation) 1∶50, anti-NeuN, (Millipore) 1∶50, and a mouse monoclonal antibody raised against whole HeV (AAHL). Bound primary antibody was detected with species-specific secondary antibodies conjugated to Alexa 488 or 568 (Invitrogen) diluted 1∶200 in PBS/BSA for 2 hours at 37°C. Sections were washed 3 times for 5 minutes with PBS and nuclei labelled with DAPI diluted 1∶1000 in dH_2_O for 30 minutes. Sections were rinsed twice with dH_2_O, mounted with Vectashield Mounting Medium (Vector Laboratories) and coverslips sealed with nail varnish.
